# Supplementary material for: Hepatoblastoma Cancer Stem Cells Express PD-L1, Reveal Plasticity and Can Emerge upon Chemotherapy
Source: Cancers (Basel). 2022 Nov 25;14(23):5825. doi: 10.3390/cancers14235825 (PMC9736435; doi:10.3390/cancers14235825)
Supplement: Supplementary file 1 [file cancers-14-05825-s001.zip › cancers-2023907-supplementary.pdf]

cancers

MDPI

Supplementary Materials

Hepatoblastoma Cancer Stem Cells Express PD-L1, Reveal Plasticity and Can Emerge Upon Chemotherapy

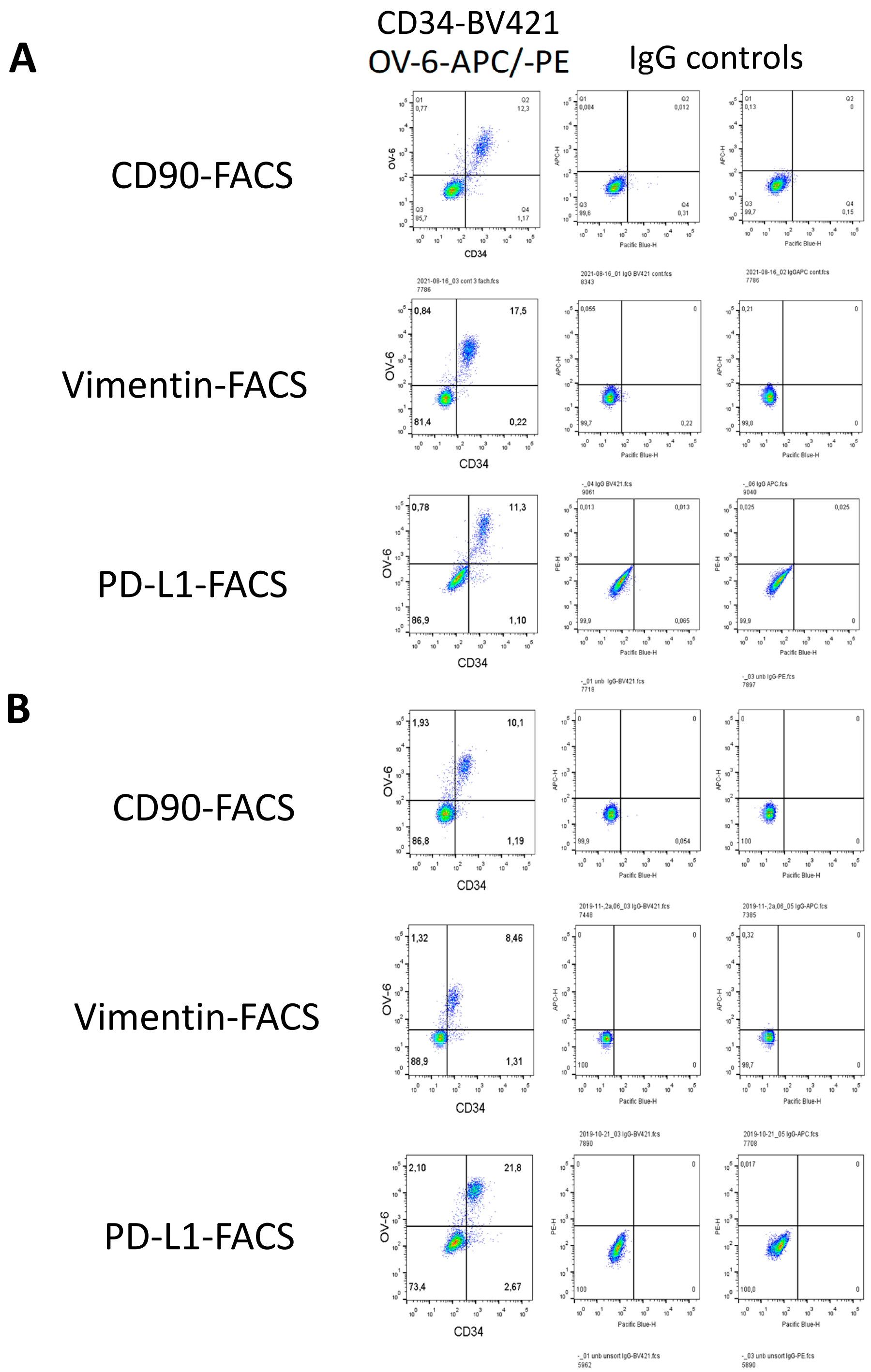

Figure S1. Background gating strategy of HuH6 and HepG2 cells. (**A**) HuH6 and (**B**) HepG2 were stained simultaneously with CD34-BV421, OV-6-APC/OV-6-PE antibodies and antibodies against a third marker (CD90-FITC, Vimentin-PE or PD-L1-APC), respectively, for flow cytometry. As controls, unspecific isotype control antibodies coupled with the according fluorescence color were used.

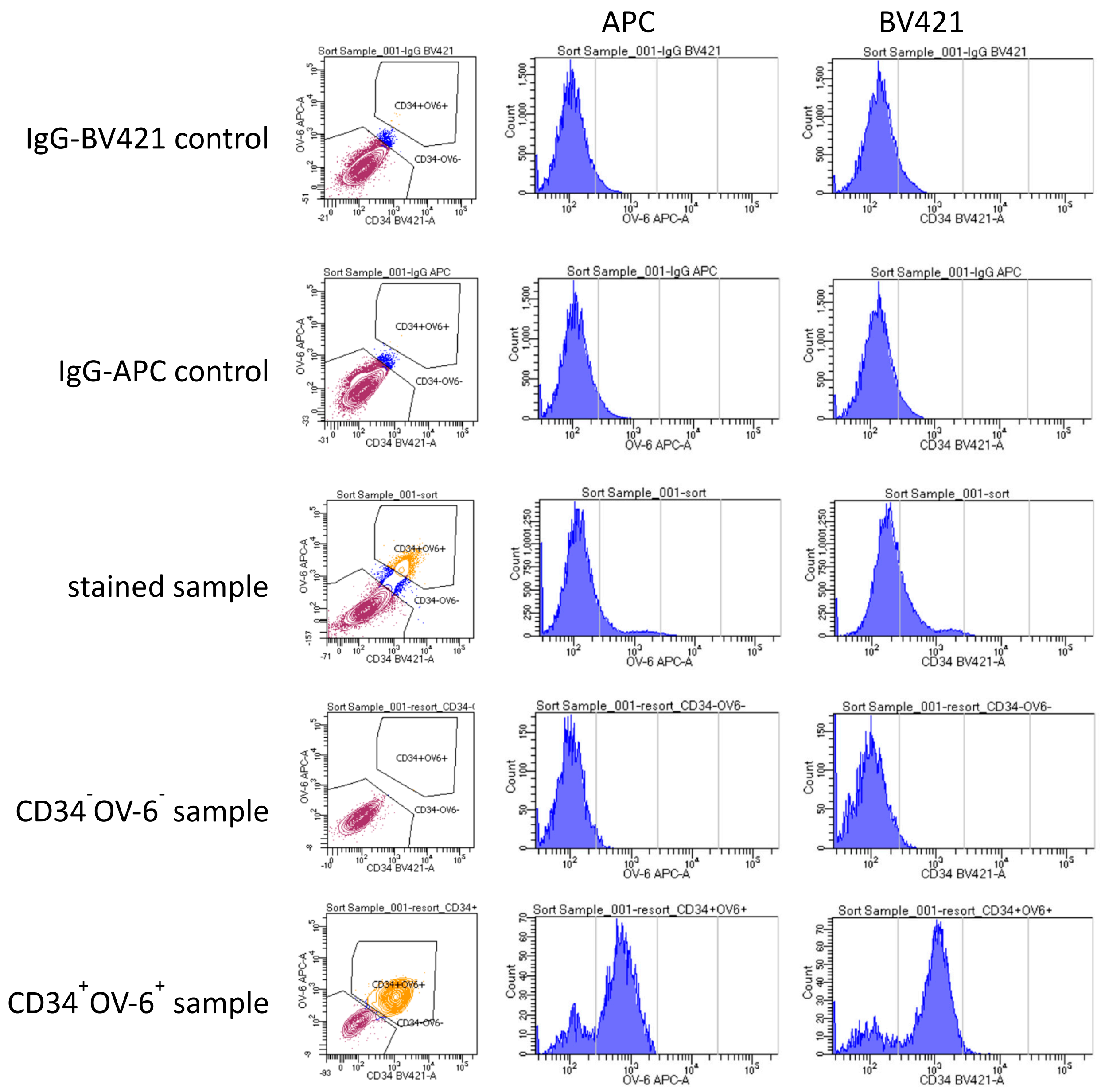

Figure S2. Background gating for HuH6 cells for FACS sort and re-analysis of sorted CD34$^-$OV-6$^-$ and CD34$^+$OV-6$^+$ cells. HuH6 cells were stained with unspecific IgG-APC and IgG-BV421 antibodies (isotype controls coupled with the according fluorescence color) and with CD34-BV421 and OV-6-APC antibodies (stained sample) and subjected to FACS sorting. After the sort, the CD34$^-$OV-6$^-$ and CD34$^+$OV-6$^+$ samples were re-analyzed.

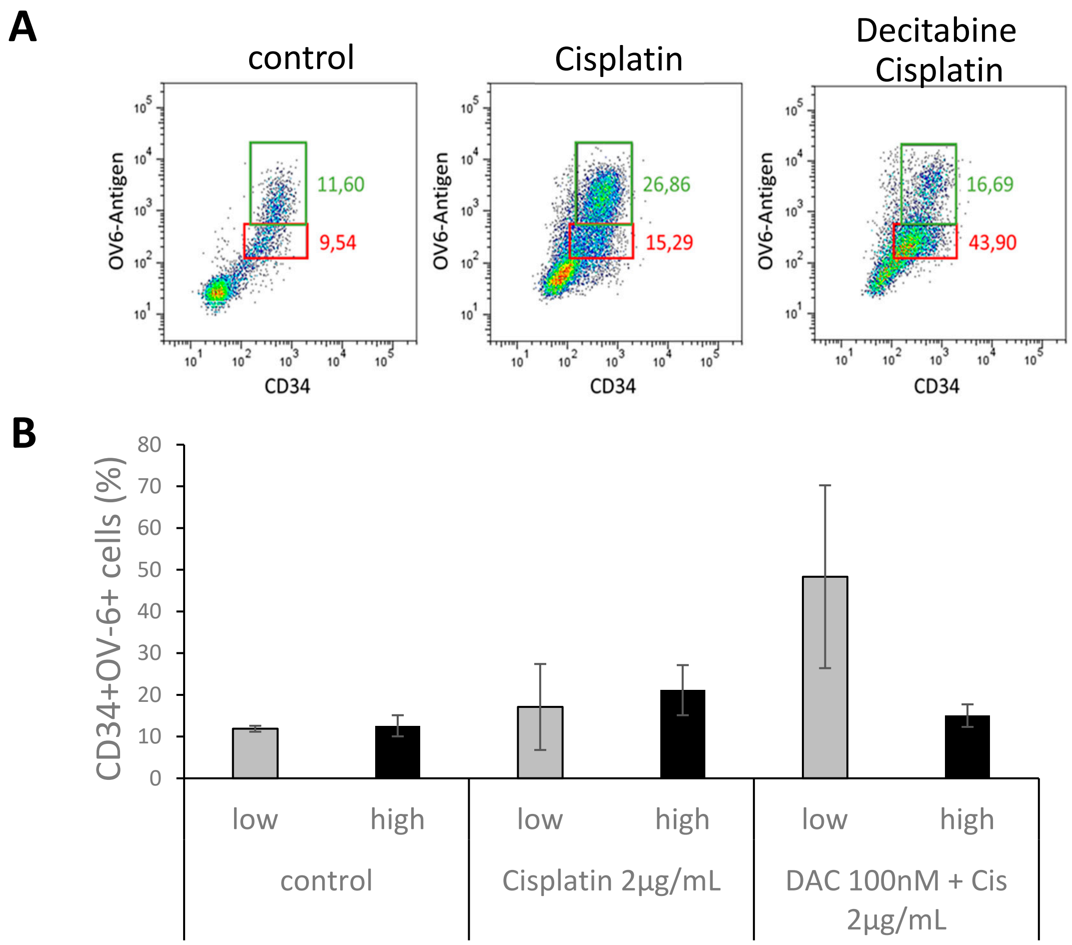

Figure S3. Combination treatment of cisplatin and decitabine results in increased CD34$^{+/low}$OV-6^{low} cells numbers. (**A**) HuH6 cells were either treated with cisplatin 2µg/mL alone or in combination with decitabine 100nM for 72h and analyzed for CD34 expression and OV-6 binding by flow cytometry. These are representative results of 3 experiments. (**B**) CD34^{low}OV-6^{low} (grey bars) and CD34^{high}OV-6^{high} (black bars) cells are presented in this histogram. The columns represent the mean with error bars depicting the standard deviation from the mean.

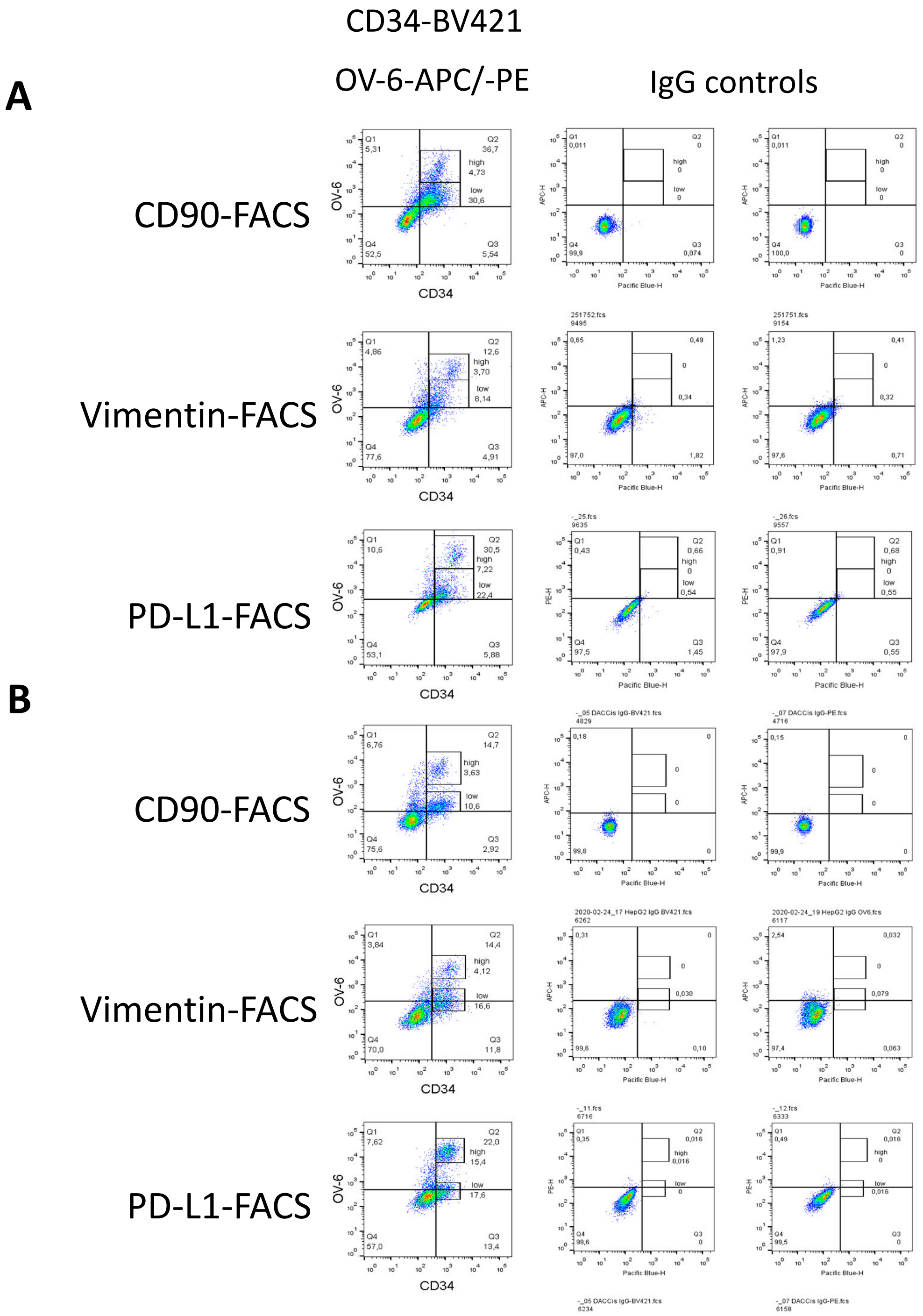

Figure S4. Background gating strategy of cisplatin and decitabine treated HuH6 and HepG2 cells. (**A**) HuH6 cells were treated with 100nM decitabine and 2µg/mL cisplatin and (**B**) HepG2 cells with 250nM decitabine and 3µg/mL cisplatin for 72h and stained simultaneously with CD34-BV421, OV-6-APC/OV-6-PE and a third marker (CD90-FITC, Vimentin-PE or PD-L1-APC), respectively, for flow cytometry. As negative controls unspecific isotype control antibodies coupled with the according fluorescence color were used.

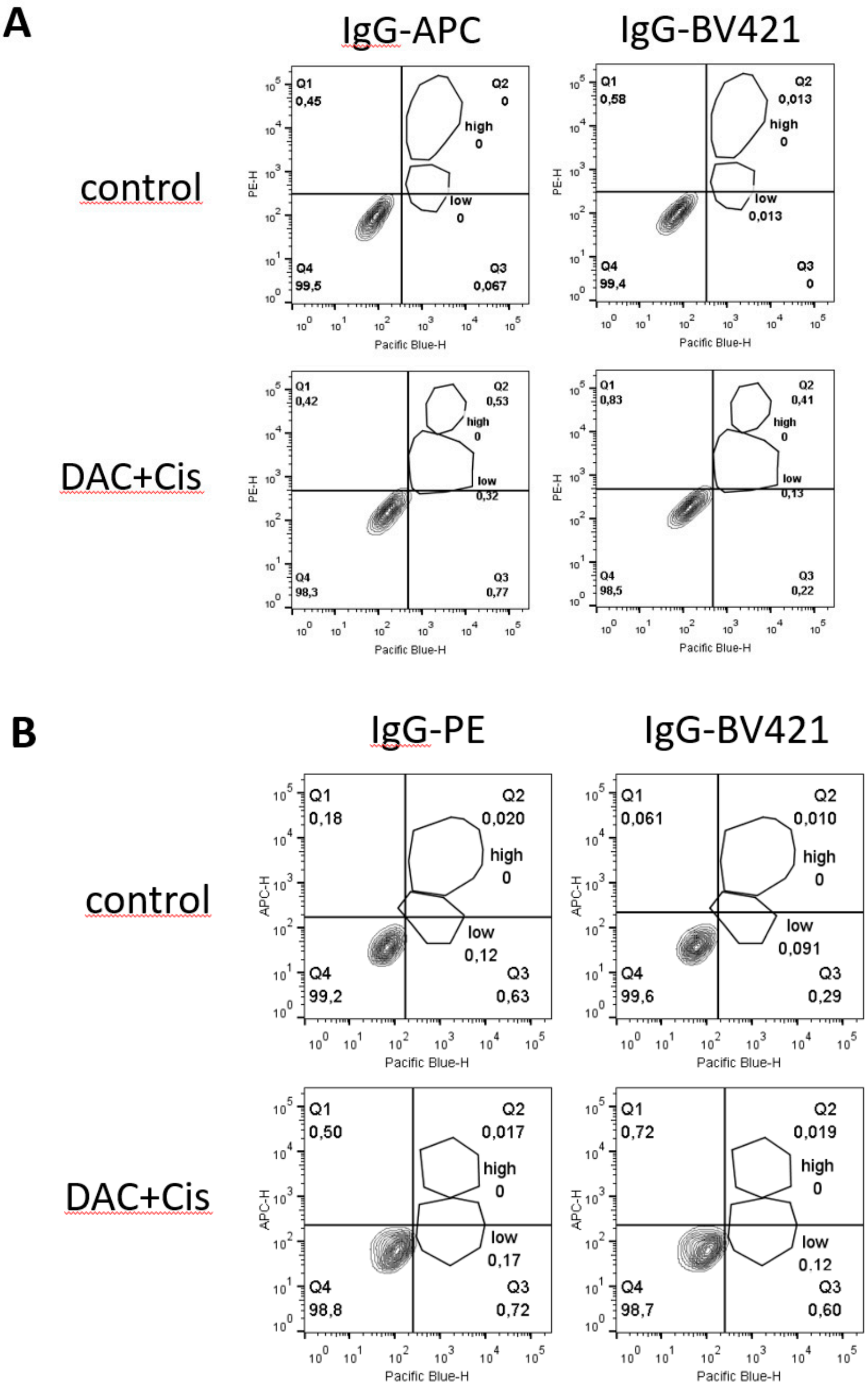

Figure S5. Background gating strategy of CD34-enriched HuH6 and HepG2 cells. (**A**) HuH6 cells were treated with 100nM decitabine and 2µg/mL cisplatin and (**B**) HepG2 cells with 250nM decitabine and 3µg/mL cisplatin for 72 h, subjected to CD34-MACS and analyzed in flow cytometry. CD34 enriched samples were stained as negative controls with unspecific isotype control antibodies coupled with the according fluorescence colors.

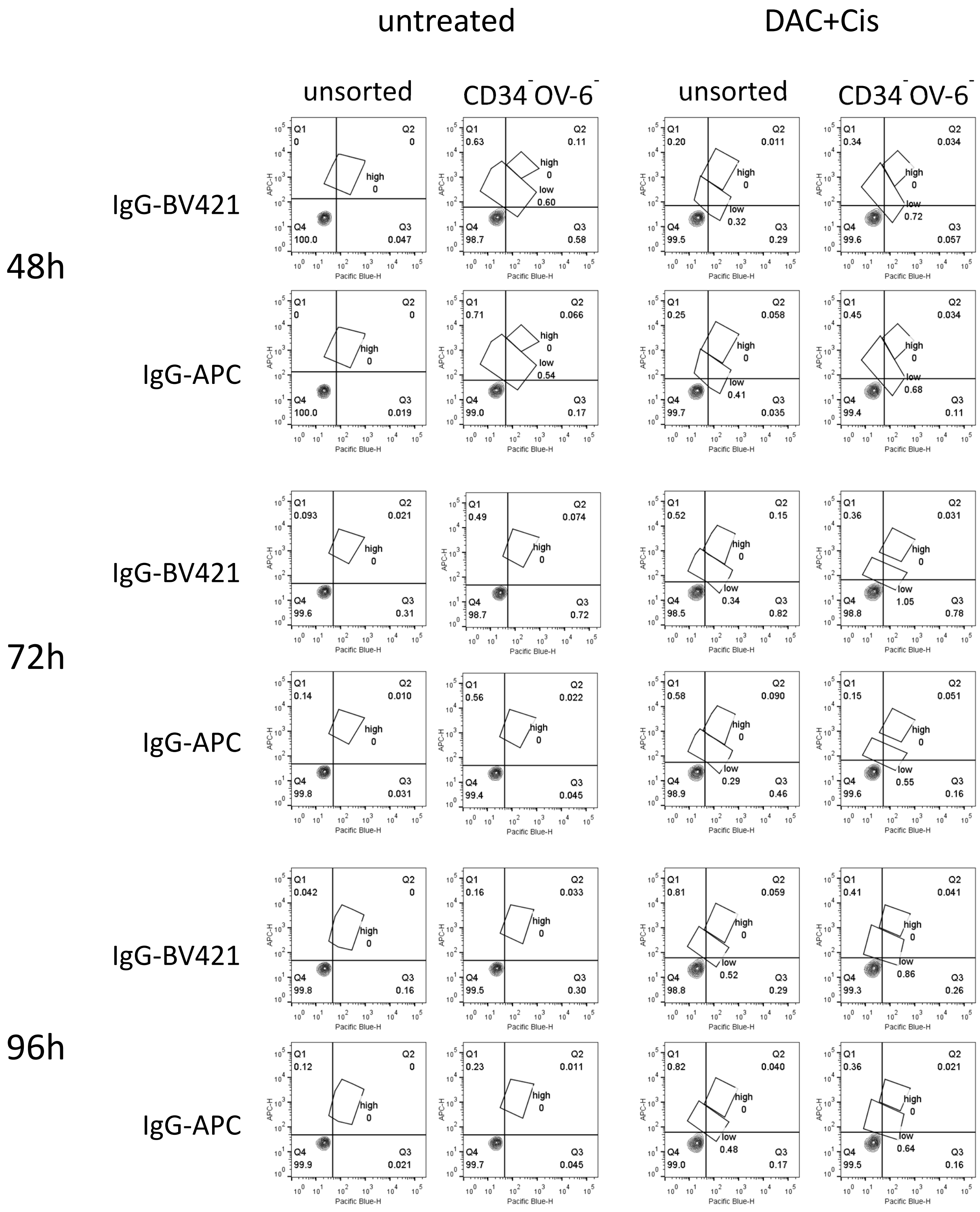

Figure S6. Background gating of cisplatin and decitabine treated CD34$^-$OV-6$^-$ sorted cells. 24 h after the FACS sort, unsorted and sorted cells were either treated with 100nM decitabine and 2µg/mL cisplatin or left untreated. Beside the staining with CD34-BV421 and OV-6-APC antibodies, the cells were also stained with unspecific isotype control antibodies coupled with BV421 or APC fluorophores over the course of 24, 48 and 72 h.

Table S1. List of qPCR primers.

Gene name	Forward primer (5′-3′)	Reverse primer (5′-3′)	Source
Oct4	GAGGCAACCTGGAGAATTTG	CGGTTACAGAACCACACTCG	NM_002701
Nanog	GAACTCTCCAACATCCTGAACC	GCGTCACACCATTGCTATTC	NM_024865
c-myc	CGGTGCAGCCGTATTTCTAC	CAGCAGCTCGAATTTCTTCC	NM_001354870
EpCAM	CGCAGCTCAGGAAGAATGTG	TGAAGTACACTGGCATTGACG	Osta et al.[1]
Albumin	TGCACAGAATCCTTGGTGAA	TTCACGAGCTCAACAAGTGC	Yang et al. [2]
SNAI1	CTCTTTCCTCGTCAGGAAGC	TAGGGCTGCTGGAAGGTAAAC	NM_005985
Twist1	CAAGCTGAGCAAGATTCAGAC C	CAGCTTGCCATCTTGGAGTC	NM_000474
CD34	CAACACCTAGTACCCTTGGAAG T	ACTGTCGTTTCTGTGATGTTTGT	Chen et al. [3]
CD90	ATCTCCTCCCAGAACGTC	ATCTCTGCACTGGAACTTG	Woeller et al. [4]
KRT14	AGAGAAGAACCGCAAGGATG	AATCTCCAGGTTCTGCATGG	NM_000526
ACTB	ACTCTTCCAGCCTTCCTTCC	TGTTGGCGTACAGGTCTTTG	NM_001101

References

1. Osta, W.A.; Chen, Y.; Mikhitarian, K.; Mitas, M.; Salem, M.; Hannun, Y.A.; Cole, D.J.; Gillanders, W.E. EpCAM is overexpressed in breast cancer and is a potential target for breast cancer gene therapy. *Cancer Res.* **2004**, *64*, 5818–5824.
2. Yang, Z.F.; Ho, D.W.; Ng, M.N.; Lau, C.K.; Yu, W.C.; Ngai, P.; Chu, P.W.K.; Lam, C.T.; Poon, R.T.P.; Fan, S.T. Significance of CD90+ cancer stem cells in human liver cancer. *Cancer Cell* **2008**, *13*, 153–166.
3. Chen, Q.; Khoury, M.; Limmon, G.; Choolani, M.; Chan, J.K.Y.; Chen, J. Human fetal hepatic progenitor cells are distinct from, but closely related to, hematopoietic stem/progenitor cells. *Stem Cells* **2013**, *31*, 1160–1169.
4. Woeller, C.F.; O'Loughlin, C.W.; Pollock, S.J.; Thatcher, T.H.; Feldon, S.E.; Phipps, R.P. Thy1 (CD90) controls adipogenesis by regulating activity of the Src family kinase, Fyn. *FASEB J.* **2015**, *29*, 920–931.
